# Heart rhythm complexity as predictors for the prognosis of end-stage renal disease patients undergoing hemodialysis

**DOI:** 10.1186/s12882-020-02196-8

**Published:** 2020-12-09

**Authors:** Hongyun Liu, Ping Zhan, Jinlong Shi, Minlu Hu, Guojing Wang, Weidong Wang

**Affiliations:** 1 Research Center for Biomedical Engineering, Medical Innovation & Research Division, Chinese PLA General Hospital, Fuxing Road, Beijing, 100853 China; 2 Medical Big Data Center, Medical Innovation & Research Division, Chinese PLA General Hospital, Fuxing Road, Beijing, 100853 China

**Keywords:** Complexity, Multiscale entropy, End-stage renal disease, Autonomic nervous system, Heart rate variability

## Abstract

**Background:**

Heart rhythm complexity, a measure of heart rate dynamics and a risk predictor in various clinical diseases, has not been systematically studied in patients with end-stage renal disease. The aim of this study is to investigate the heart rhythm complexity and its prognostic value for mortality in end-stage renal disease patients undergoing hemodialysis.

**Methods:**

To assess heart rhythm complexity and conventional heart rate variability measures, 4-h continuous electrocardiography for a retrospective cohort of 202 ostensibly healthy control subjects and 51 hemodialysis patients with end-stage renal disease were analyzed. Heart rhythm complexity was quantified by the complexity index from the measurement of the multiscale entropy profile.

**Results:**

During a follow-up of 13 months, 8 people died in the patient group. Values of either traditional heart rate variability measurements or complexity indices were found significantly lower in patients than those in healthy controls. In addition, the complexity indices (Area 1–5, Area 6–15 and Area 6–20) in the mortality group were significantly lower than those in the survival group, while there were no significant differences in traditional heart rate variability parameters between the two groups. In receiver operating characteristic curve analysis, Area 6–20 (AUC = 0.895, *p* < 0.001) showed the strongest predictive power between mortality and survival groups.

**Conclusion:**

The results suggest that heart rhythm complexity is impaired for patients with end-stage renal disease. Furthermore, the complexity index of heart rate variability quantified by multiscale entropy may be a powerful independent predictor of mortality in end-stage renal disease patients undergoing hemodialysis.

## Background

End-stage renal disease (ESRD), characterized by permanent loss of renal function, is the final stage in chronic kidney disease that becomes a global health problem with increasing prevalence [1]. Despite recent progress in medical management and dialysis therapy technique, mortality rates remain high in ESRD patients undergoing hemodialysis. The major causes of ESRD are largely the same causes that contribute to cardiovascular disease [2]. In addition, sudden cardiac death is common in patients receiving hemodialysis and may account for about 30% of overall mortality, which could partly owe to the impairment of the autonomic nervous system (ANS) [3–5]. Identification of high-risk patients with ESRD is of clinical importance and considerable practical value in strengthening their treatment strategies.

Patients with ESRD undergoing chronic hemodialysis is usually present a dysfunction of the ANS, exhibited as low parasympathetic/vagal modulation associated with high sympathetic modulation of the heart rate assessed by heart rate variability (HRV) analysis [6–8]. Several previous studies have used HRV parameters based on traditional linear HRV analysis to predict patient outcomes and found that lower HRV is related to an increased risk of cardiovascular and all-cause mortality in patients with ESRD receiving chronic hemodialysis [9–13]. However, the regulation of heart rate by the ANS is considered to be a non-linear physiological process with complexity. Recently, multiscale entropy (MSE) measures quantify multiple spatial and temporal scale complexity of heart rate dynamics, have been introduced as new risk stratifiers [14, 15]. At present, MSE has been extensively applied in the assessment of heart rhythm complexity in heart failure, stroke, myocardial infarction, epilepsy and other diseases for predicting patient outcomes [16–19]. Previous studies have also demonstrated that MSE parameters have greater predictive power than conventional HRV measures for risk stratification and prognosis [19, 20]. Heart rhythm complexity impairment has been associated with adverse outcomes in clinical settings. However, its potential relation to the increased risk of death in ESRD patients undergoing hemodialysis may be underappreciated and need to be further elucidated. Therefore, the aim of the present study was to investigate the heart rate complexity derived from MSE analysis of long-term electrocardiograms (ECG) and its prognostic value for all-cause mortality in patients with ESRD receiving hemodialysis therapy.

## Methods

### The study protocol and population

Healthy database (THEW identification: E-HOL-03-0202-003) with 202 24-h ECG recordings and ESRD database (THEW identification: E-HOL-03-0202-016) contains 51 48-h long-term ECG recordings were selected from the Telemetric and Holter ECG Warehouse (THEW, http://thew-project.org/datab ases.htm) at University of Rochester. Raw ECG data, as well as automated beat annotations reviewed and adjudicated manually, are available in both databases. All human data were obtained retrospectively from completed, Institutional-Review-Board-approved clinical research studies with subject de-identification. These trials complied with the Declaration of Helsinki and all subjects signed informed consent documents.

The ESRD database comprises Holter recordings with a sampling frequency of 1000 Hz from 51 ESRD patients with a high risk of death. All 51 ESRD patients underwent high-resolution 12-lead 48-h continuous ECG monitoring, and THEW provides 12-lead raw ECG and heartbeat interval data. ESRD subjects receiving hemodialysis confirmed the history of diabetes or hypertension requiring treatment entered into this study. The ESRD patients were enrolled from February 13, 2009 to June 18, 2010, and they completed their 13-month follow-up evaluation. All patients Exclusion criteria included a history of chronic atrial fibrillation, with class I antiarrhythmic, pacemaker, implantable cardioverter-defibrillator device, cardiac resynchronization therapy device, female subject of childbearing potential not using medically prescribed contraceptive measures and subject unable to cooperate with the protocol due to dementia, psychological, or other related reason. The Healthy database comprises 24-h Holter recordings from 202 ostensibly healthy subjects. Subjects had (1) no overt cardiovascular disease or history of cardiovascular disorders; (2) no reported medications, history of high blood pressure and chronic illness, (3) a normal physical examination, (4) a 12-lead ECG showing sinus rhythm with normal waveforms (or a normal echocardiogram and normal ECG exercise testing in the presence of any questionable findings ECG changes). The ECG signals were recorded at a sampling frequency of 200 Hz. In order to reduce the influence of gender and age on ECG parameters, we try to match each ESRD patient with a healthy control with the same gender and close age. After excluding the poor signal quality and incomplete ECG recordings, 51 ostensibly age-matched healthy control subjects were selected from 202 healthy subjects and eventually enrolled in the present study.

### ECG preprocessing

All long-term ECG recordings were analyzed with Kubios (Kubios 2.2, University of Eastern Finland, Kuopio), on which R waves were detected and labeled automatically. Heart-beat interval between 300 and 2000 ms, consecutive heart-beat interval differences ≤200 ms, and prolongations or shortenings ≤20% than the average of five preceding sinus rhythm heart-beat intervals were considered as sinus rhythm QRS complexes [21]. Thereafter, automatic annotated results were carefully visual inspected and manually corrected by editing ectopic beats, arrhythmias and noise to suppress computational errors. Four-hour episodes of heart-beat intervals without naps and exercise within daytime (between 8 a.m. and 5 p.m.) were extracted from each recording for MSE and traditional HRV analysis [18, 19]. The 4-h ECG recordings of ESRD patients used in the present study were selected after the ideal body weight to reduce the influence of volume overload on HRV. All ECG segments were selected from the same period to reduce the confounding effects that may occur due to sleep or diurnal rhythm. Furthermore, four-hour ECG segments of patients with ESRD after hemodialysis sessions while performing their usual daily activities were used for further analysis.

### Traditional HRV analysis

Traditional techniques of HRV analysis are grouped into the time domain, frequency domain, and non-linear methods. The time-domain measures including mean heartbeat intervals (Mean RR), standard deviation of the heartbeat intervals (SDNN), square root of the mean of sum of squares of the differences between adjacent heartbeat intervals (RMSSD), and percentage of the absolute change in consecutive heartbeat interval exceeds 50 ms (pNN50) were calculated to represent the total variance and vagal modulation of heart rate [22]. Based on the Fast Fourier transform spectrum, the frequency domain measures were computed from the power spectral density estimate for each frequency band including absolute power values of very low frequency (VLF, 0.0033–0.04 Hz), low frequency (LF, 0.04–0.15 Hz), high frequency (HF, 0.15–0.40 Hz) bands, total power (TP, 0.0033–0.40 Hz) and LF/HF power ratio [22]. The VLF, LF, HF and TP were also transformed in natural logarithmic (ln) value. Five traditional non-linear measures were also taken into consideration to characterize the properties of HRV. SD1 denotes the short-term variability caused by respiration, whereas SD2 denotes the long-term variability with both calculated through the Poincaré plot method [23]. Approximate entropy (ApEn) quantifies the single-scale complexity or regularity of the HRV time series by measuring the unpredictability of fluctuation patterns, and more uncorrelated random HRV signals usually produce higher ApEn value [24]. As a technique for characterizing the nature of long-range correlations in time series, detrended fluctuation analysis (DFA) was applied in the present study to quantify slope α1 and α2 for characterizing the short-term and long-term fluctuations of HRV signal, respectively [25].

### MSE analysis

The MSE technique was proposed to characterize complex structure of non-linear and non-stationary physiological signals in different temporal scales that ignored by traditional entropy methods. It comprises of two steps: 1) coarse-graining the time series in finite length into different time scales; 2) quantifying the degree of irregularity in each coarse-grained time series by sample entropy calculations [14, 15]. The quantified entropy values of coarse-grained time series then are represented as the function of time scale factors to evaluate the complex structure of physiological time series, and the features of the MSE curve can be extracted for clinical categorization in several diseases [16–19]. In-depth, details of this methodology have been previously described [14, 15]. In the present study, the complexity index (CI) of the HRV time series were quantified by curve fitting and calculating the area between the MSE curve and the axis of scale factors [18, 19]. The linear-fitted slope (Slope 5) and the area under the MSE curve between scales 1 and 5 (Area 1–5) were calculated to quantify the short-term complexity and to characterize the short-scale modulation pattern. Long time scale complexity was quantified by the fitted area under the MSE curve between scales 6 and 15 (Area 6–15) and between scales 6 and 20 (Area 6–20), respectively. Since low frequency drifts, high frequency non-stationarities and general hidded trends longer than 2 h may lead to incorrectly increased irregularity and diminished sequence matching manifesting unpredictable effects on calculated sample entropy values. Empirical mode decomposition (EMD) is suitable for decomposition of non-stationary, non-linear physiologic time series and possesses advantages over wavelet and Fourier analysis because it employs a fully adaptive approach derived by means of a sifting process. In order to remove such effects, we used empirical mode decomposition (EMD) method for raw HRV time series filtering before performing MSE [18, 19].

### Statistical analysis

Clinical data and parameters of ECG recordings were presented as median (25th and 75th percentiles). Gaussian distribution and homogeneity of variance tests were applied to determine the distribution and homoscedasticity of sample data. As a result of the non-normal distribution and heterogeneity of variance of some sample data, continuous variables were compared between different groups by the Mann-Whitney U test. For single predictive variable analysis using qualitative or categorical variables, Fisher’s exact tests were applied for comparison between different groups. Correlations between clinical variables and independent factors that predicting all-cause death for ESRD patients were performed using Spearman’s correlation tests. The receiver operating characteristics (ROC) curve was created based on the sensitivity and specificity of HRV measures in predicting all-cause death in ESRD patients undergoing hemodialysis. The area under the ROC curve (AUC) gave an estimate of the overall discriminate ability (AUC = 0.5 indicates no discrimination and an AUC = 1.0 indicates a perfect diagnostic test). Statistical analyses were performed using SPSS version 20 software package (SPSS, Chicago, Ill, USA). The maximal hazards ratio and independent correlation of variables with mortality was determined by Cox regression analysis. Then, Kaplan-Meier event probability curves and log rank analysis of the dichotomized groups were obtained. For all statistical analysis, *p* values were corrected by the false discovery rate (FDR) method for multiple comparisons and *p* < 0.05 was considered significant.

## Results

### Study population

Eventually, a total of 51 ESRD patients and 51 ostensibly healthy control subjects were enrolled in the present study. After a follow-up period of around 13 months, 8 (15.7%) patients died in the ESRD group. There were 4 cardiac-related deaths and the other 4 patients died for unknown reasons. As shown in Table 1, ideal body weight, body mass index (BMI) and pre-dialysis systolic blood pressure (BP_SYS) values were significantly higher in ESRD patients while the prevalence of smoking (11.8% vs. 39.2%, *p* < 0.001) was lower in ESRD group. However, there were no significant differences in gender, age, height and pre-dialysis diastolic blood pressure (BP_DIA) between the ESRD and healthy groups. Detail information on demographic data, clinical characteristics, and laboratory data prior to hemodialysis for ESRD patients including both survival and mortality groups are presented in Table 2. No clinical variable was significantly different between these two groups (survival and mortality).

**Table 1:** Demographic data and basic characteristics of ESRD patients and healthy control subjects

Variables	ESRD patients (***n*** = 51)	Healthy control subjects (***n*** = 51)	***P*** value
Male/female	30/21	24/27	0.321
Age (year)	57 (52–67)	58 (52–65)	0.859
Height (cm)	169 (162–177)	168 (159–175)	0.451
Weight (kg)	82 (72–97)	70 (64–83)	< 0.001
BMI (kg/m^2^)	29.37 (24.39–33.47)	25.30 (23.62–27.48)	< 0.001
BP_SYS (mmHg)	143 (127–169)	120 (118–140)	< 0.001
BP_DIA (mmHg)	73 (65–88)	80 (75–80)	0.227
Smoking, n (%)	6 (11.8)	20 (39.2)	0.003
Diabetes Mellitus, n(%)	32 (62.7%)	N.A	N.A
Hypertension, n(%)	51 (100%)	N.A	N.A
LVEF (%)	60 (55–65)	N.A	N.A
Duration of dialysis (year)	2 (3–6)	N.A	N.A
Kt/V	1.42 (1.24–1.61)	N.A	N.A
URR (%)	74 (70–79)	N.A	N.A
nPCR	0.85 (0.72–0.97)	N.A	N.A
Sodium (mEq/dL)	139 (137–141)	N.A	N.A
Potassium (mEq/dL)	5.1 (4.4–5.4)	N.A	N.A
Chloride (mEq/dL)	100 (97–103)	N.A	N.A
Bicarbonite (mEq/dL)	26 (22–29)	N.A	N.A
Blood Urea Nitrogen (mg/dL)	58 (45–72)	N.A	N.A
Creatinine (mg/dL)	8.3 (6.6–10.4)	N.A	N.A
Glucose (mg/dL)	106 (88–137)	N.A	N.A
Calcium (mg/dL)	8.7 (8.3–9.2)	N.A	N.A
Phosphate (mg/dL)	5.3 (4.3–6.2)	N.A	N.A
Calcium Phosphate Product	44.7 (35.6–51.1)	N.A	N.A
Albumin (g/dL)	4.0 (3.8–4.2)	N.A	N.A
Hemoglobin (g/dL)	11.7 (11.0–12.3)	N.A	N.A
Hematocrit (%)	36.0 (34.0–38.0)	N.A	N.A
β-blocker, n (%)	34 (66.7)	N.A	N.A
CCB, n (%)	25 (49.0)	N.A	N.A
ACEI, n (%)	15 (29.4)	N.A	N.A

*ESRD* end-stage renal disease, *BMI* body mass index, *BP_SYS* systolic blood pressure, *BP_DIA* diastolic blood pressure, *N.A* not applicable, *LVEF* left ventricular ejection fraction, *URR* urea reduction ratio, *nPCR* normalized protein catabolic rate, *CCB* calcium channel blocker, *ACEI* angiotensin-converting-enzyme inhibitor

**Table 2:** Clinical data of the ESRD patients in survival and mortality groups

Variables	Survival (***n*** = 43)	Mortality (***n*** = 8)	***P*** value
Male/female	25/18	5/3	1.000
Age (year)	55 (52–65)	66 (62–70)	0.087
Height (cm)	170 (162–178)	168 (166–171)	0.595
Weight (kg)	83 (72–101)	76 (65–93)	0.223
BMI (kg/m^2^)	30.22 (24.39–34.29)	26.28 (24.09–31.76)	0.214
BP_SYS (mmHg)	141 (127–169)	146 (123–183)	0.866
BP_DIA (mmHg)	77 (69–83)	71 (58–88)	0.517
Smoking, n (%)	5 (11.6)	1 (12.5)	1.000
Diabetes Mellitus, n (%)	25 (58.1)	7 (87.5)	0.672
Hypertension, n (%)	43(100)	8 (100)	1.000
Duration of dialysis (year)	3 (2–6)	3 (2–7)	0.793
LVEF (%)	60 (55–64)	62 (51–85)	0.370
Kt/V	1.42 (1.24–1.61)	1.44 (1.25–1.64)	0.776
URR (%)	74 (70–79)	75 (69–80)	0.640
nPCR	0.85 (0.69–0.96)	0.95 (0.76–1.05)	0.158
Sodium (mEq/dL)	139 (137–141)	139 (137–142)	0.886
Potassium (mEq/dL)	5.0 (4.4–5.3)	5.2 (4.5–5.5)	0.357
Chloride (mEq/dL)	100 (97–103)	100 (97–103)	0.897
Bicarbonite (mEq/dL)	27 (23–29)	22 (21–25)	0.065
Blood Urea Nitrogen (mg/dL)	56 (43–70)	70 (50–89)	0.214
Creatinine (mg/dL)	8.3 (6.6–9.5)	9.0 (6.6–11.0)	0.595
Glucose (mg/dL)	106 (88–143)	110 (81–136)	0.698
Calcium (mg/dL)	8.8 (8.4–9.2)	8.6 (6.8–9.1)	0.232
Phosphate (mg/dL)	5.2 (3.9–6.0)	5.6 (4.8–6.6)	0.249
Calcium Phosphate Product	44.5 (35.5–51.3)	46.0 (42.0–49.7)	0.679
Albumin (g/dL)	4.0 (3.8–4.2)	4.0 (3.5–4.3)	0.630
Hemoglobin (g/dL)	11.7 (10.9–12.3)	11.6 (11.2–12.7)	0.688
Hematocrit (%)	36.0 (33.0–38.0)	37.0 (36.0–39.0)	0.076
β-blocker, n (%)	29 (67.4)	5 (62.5)	0.541
CCB, n (%)	20 (46.5)	5 (62.5)	0.329
ACEI, n (%)	13 (30.2)	2 (25.0)	0.565

*ESRD* end-stage renal disease, *LVEF* left ventricular ejection fraction, *URR* urea reduction ratio, *nPCR* normalized protein catabolic rate, *CCB* calcium channel blocker, *ACEI* angiotensin-converting-enzyme inhibitor

### Holter data

**Fig. 1:**
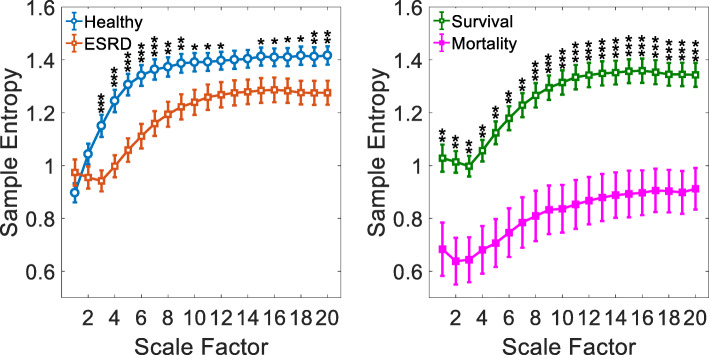
The sample entropy over different time scales. The orange square open symbols represented the entropy of patients with ESRD, and the light blue open circles the entropy of healthy control subjects. The green open squares represented the entropy of the survival group after session hemodialysis treatment, and the pink solid squares the entropy of the mortality group after session hemodialysis treatment. Symbols represent the mean values of entropy for each group and bars represent the standard error ($SE= SD/\sqrt{n}$, where n is the number of subjects). *p < 0.05, **p < 0.01, and ***p < 0.001 for comparison between groups

The results of traditional HRV and MSE analyses in ESRD and healthy groups are presented in Table 3 and Fig. 1. For traditional HRV parameters and CI derived from the MSE profiles, ESRD patients had significantly lower SDNN, RMSSD, pNN50, VLF, LF, HF, TP, LF/HF, SD1, SD2, α1, Slope 5, Area 1–5, Area 6–15 and Area 6–20 in comparison to the healthy control subjects. In contrast, patients with ESRD had significantly higher ApEn and α2 values than those of healthy controls (all *p* < 0.05, Table 3). The ESRD patients exhibited significantly reduced entropy values over most of the time scales, except scale 1, 2, 13 and 14 (Fig. 1). In addition, all of the average MSE curves of ESRD group had a pattern of an initial decrease which is different from that of the healthy control group.

**Table 3:** Traditional HRV measurements and CI over 4-h continuous ECG recordings for ESRD patients and healthy control subjects

Variables	ESRD patients (***n*** = 51)	Healthy control subjects (***n*** = 51)	***P*** value
**Traditional HRV analysis**			
Mean RR (msec)	779 (733–858)	754 (690–836)	0.137
SDNN (msec)	36 (28–50)	71 (59–95)	< 0.001
RMSSD (msec)	12 (8–15)	18 (13–29)	< 0.001
pNN50 (%)	0.14 (0.02–1.06)	1.77 (0.42–6.64)	< 0.001
VLF (msec^2^)	18 (10–41)	68 (45–142)	< 0.001
LF (msec^2^)	50 (23–178)	333 (234–790)	< 0.001
HF (msec^2^)	34 (14–65)	106 (46–270)	< 0.001
TP (msec^2^)	109 (55–280)	511 (376–1232)	< 0.001
lnVLF (msec^2^)	2.88 (2.32–3.69)	4.23 (3.85–4.84)	< 0.001
lnLF (msec^2^)	3.91 (3.17–5.17)	5.81 (5.46–6.61)	< 0.001
lnHF (msec^2^)	3.51 (2.68–4.13)	4.67 (3.86–5.48)	< 0.001
lnTP (msec^2^)	4.69 (4.10–5.62)	6.24 (5.94–7.11)	< 0.001
LF/HF	1.68 (0.84–3.96)	3.42 (2.37–6.85)	< 0.001
SD1 (msec)	8 (5–10)	12 (9–20)	< 0.001
SD2 (msec)	14 (10–23)	31 (26–47)	< 0.001
ApEn	1.47 (1.36–1.52)	1.36 (1.27–1.46)	< 0.001
α1	1.07 (0.83–1.26)	1.32 (1.15–1.49)	< 0.001
α2	0.62 (0.49–0.71)	0.52 (0.46–0.61)	0.005
**MSE analysis**			
Slope 5	0.04 (−0.04–0.08)	0.10 (0.07–0.15)	< 0.001
Area 1–5	3.90 (3.29–4.49)	4.39 (3.95–5.24)	0.004
Area 6–15	11.62 (9.13–12.85)	12.63 (11.42–13.65)	0.013
Area 6–20	18.10 (14.34–20.28)	19.79 (18.19–21.70)	0.017

*ESRD* end-stage renal disease, *HRV* heart rate variability, *Mean RR* mean heartbeat intervals, *SDNN* standard deviation of the heartbeat intervals, *RMSSD* square root of the mean of sum of squares of the differences between adjacent heartbeat intervals, *pNN50* percentage of the absolute change in consecutive heartbeat interval exceeds 50 ms, *VLF* very low frequency, *LF* low frequency, *HF* high frequency, *TP* total power, *ApEn* approximate entropy, *MSE* multiscale entropy

For all the analyzed traditional HRV analyses, there were no significant differences between the survival and mortality groups (all *p* > 0.05, Table 4). For MSE analysis, the entropy values were significantly lower over different time scales in the mortality group (Fig. 1). Meanwhile, the mortality group had significantly lower CI including Area 1–5 (2.49 (1.79–3.68) vs. 4.11 (3.36–4.72), *p* = 0.002), Area 6–15 (7.39 (6.27–9.92) vs. 11.92 (10.16–13.69), *p* < 0.001) and Area 6–20 (11.96 (9.84–15.54) vs. 18.78 (16.63–21.60), p < 0.001) than those of survival group (Table 4). Furthermore, the above independent risk factors or predictors derived from MSE quantification did not significantly correlate with any clinical variables observed in the study (all *p* > 0.05, Table 5).

**Table 4:** Traditional HRV measurements and CI over 4-h continous ECG recordings for the study subjects in survival and mortality groups

Variables	Survival (***n*** = 43)	Mortality (***n*** = 8)	***P*** value
**Traditional HRV analysis**			
Mean RR (msec)	779 (732–885)	795 (753–852)	0.846
SDNN (msec)	34 (26–48)	43 (35–53)	0.143
RMSSD (msec)	13 (8–15)	9 (6–19)	0.445
pNN50 (%)	0.14 (0.02–1.06)	0.07 (0.01–3.97)	0.917
VLF (msec^2^)	21 (10–51)	11 (4–22)	0.117
LF (msec^2^)	53 (25–183)	29 (11–70)	0.344
HF (msec^2^)	34 (16–65)	16 (7–102)	0.526
TP (msec^2^)	141 (70–311)	67 (32–155)	0.271
lnVLF (msec^2^)	3.02 (2.36–3.82)	2.43 (1.76–2.79)	0.119
lnLF (msec^2^)	3.96 (3.23–5.20)	3.37 (2.77–4.07)	0.351
lnHF (msec^2^)	3.52 (2.85–4.10)	2.76 (2.31–4.26)	0.533
lnTP (msec^2^)	4.95 (4.25–4.95)	4.18 (3.71–4.98)	0.277
LF/HF	1.92 (0.85–3.97)	1.41 (0.46–3.46)	0.460
SD1 (msec)	9 (5–10)	6 (4–14)	0.476
SD2 (msec)	15 (10–23)	11 (7–16)	0.344
ApEn	1.47 (1.36–1.52)	1.45 (1.36–1.49)	0.430
α1	1.07 (0.83–1.27)	1.04 (0.57–1.21)	0.476
α2	0.64 (0.52–0.71)	0.59 (0.34–0.73)	0.445
**MSE analysis**			
Slope 5	0.05 (0.04–0.09)	0.02 (0.05–0.07)	0.509
Area 1–5	4.11 (3.36–4.72)	2.49 (1.79–3.68)	0.002
Area 6–15	11.92 (10.16–13.69)	7.39 (6.27–9.92)	< 0.001
Area 6–20	18.78 (16.63–21.60)	11.96 (9.84–15.54)	< 0.001

*HRV* heart rate variability, *Mean RR* mean heartbeat intervals, *SDNN* standard deviation of the heartbeat intervals, *RMSSD* square root of the mean of sum of squares of the differences between adjacent heartbeat intervals, *pNN50* percentage of the absolute change in consecutive heartbeat interval exceeds 50 ms, *VLF* very low frequency, *LF* low frequency, *HF* high frequency, *TP* total power, *ApEn* approximate entropy, *MSE* multiscale entropy

**Table 5:** Correlations between MSE parameters and clinical characteristics

Variables	ESRD patients (***n*** = 51)		
	Area 1–5	Area 6–15	Area 6–20
Age (year)	0.061	−0.024	−0.050
BP_SYS (mmHg)	−0.100	−0.152	−0.133
BP_DIA (mmHg)	−0.080	0.039	0.055
BMI (kg/m^2^)	0.017	−0.152	−0.134
LVEF (%)	−0.058	−0.061	− 0.069
Duration of dialysis (year)	0.112	0.213	0.235
Kt/V	0.022	−0.045	−0.038
URR (%)	0.222	0.038	0.009
nPCR	−0.183	−0.219	− 0.219
Sodium (mEq/dL)	0.096	0.083	0.099
Potassium (mEq/dL)	−0.163	−0.128	− 0.113
Chloride (mEq/dL)	−0.121	− 0.005	0.005
Bicarbonite (mEq/dL)	0.197	0.106	0.111
Blood Urea Nitrogen (mg/dL)	−0.172	−0.081	− 0.060
Creatinine (mg/dL)	0.039	0.068	0.072
Glucose (mg/dL)	−0.130	−0.129	− 0.117
Calcium (mg/dL)	0.102	0.183	0.165
Phosphate (mg/dL)	−0.049	−0.131	− 0.138
Calcium Phosphate Product	0.019	−0.098	−0.112
Albumin (g/dL)	0.118	0.087	0.071
Hemoglobin (g/dL)	−0.018	−0.075	− 0.080
Hematocrit (%)	−0.045	− 0.232	−0.243

Values are correlation coefficients; ***p < 0.001; **p < 0.01; *p < 0.05. *ESRD* end-stage renal disease, *BMI* body mass index, *BP_SYS* systolic blood pressure, *BP_DIA* diastolic blood pressure, *LVEF* left ventricular ejection fraction, *URR* urea reduction ratio, *nPCR*normalized protein catabolic rate

**Fig. 2:**
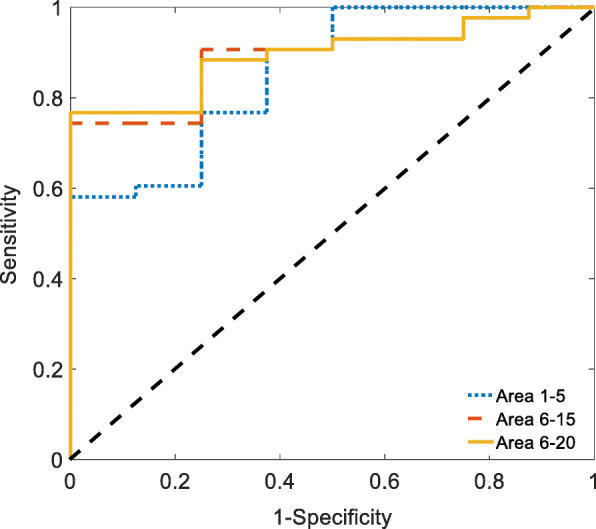
Analysis of the discrimination power of the survival and mortality by receiver operating characteristic (ROC) curve analysis. The areas under the curve of Area 1–5, Area 6–15 and Area 6–20 were 0.858, 0.892 and 0.895, respectively

**Fig. 3:**
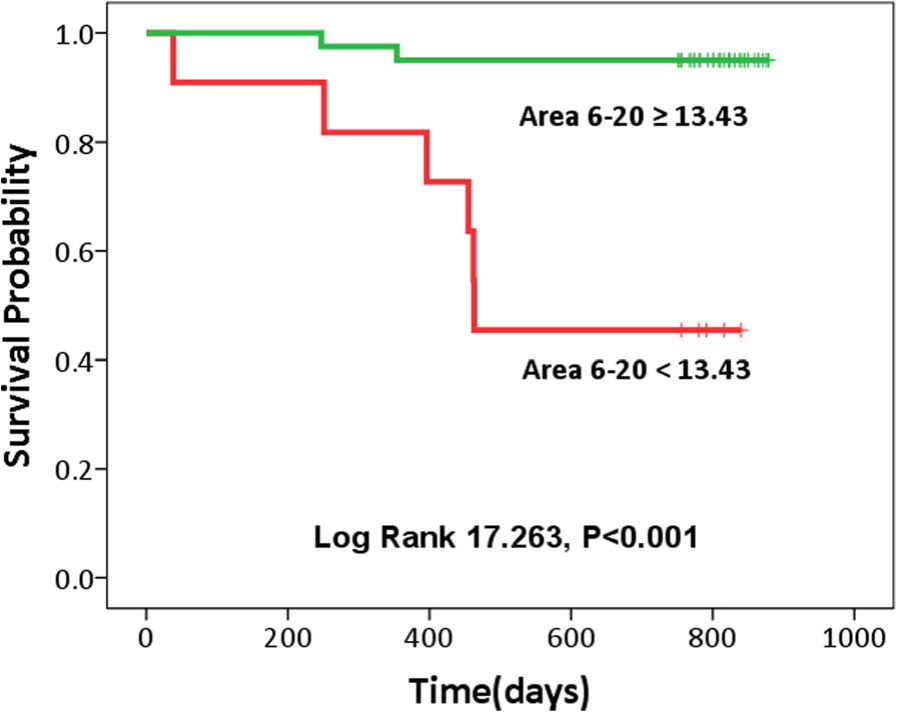
Kaplan-Meier survival curves (*P* < 0.001) for all-cause mortality according to the the MSE parameters Area 6–20. The mortality for Area 6–20 ≥ 13.43 and Area 6–20 < 13.43 were 4.80 and 66.7%, respectively

The ROC curves of the predictive parameters (Area 1–5, Area 6–15 and Area 6–20) were depicted in Fig. 2. Area 6–20 (AUC = 0.895) showed the best overall discriminative power than Area 1–5 (AUC = 0.858) and Area 6–15 (AUC = 0.892) in the outcome prediction for ESRD patients receiving hemodialysis. Figure 3 showed Kaplan-Meier survival curves for all-cause death according to the contribution of Area 6–20. For the ESRD patients undergoing hemodialysis, survival seems to be related to whether the values of Area 6–20 were low (< 13.43). Cox regression analysis demonstrated that ESRD patients with Area 6–20 < 13.43 had a higher risk of death (4.8% vs. 66.7%) than those with Area 6–20 ≥ 13.43 (Hazard ratio = 13.61, 95% CI: 2.74–67.77). However, no statistical risk factors were found in data of demographic characteristics, baseline clinical evaluation, or laboratory tests. The Area 6–20 may be a significant independent predictor of all-cause mortality for patients with ESRD, and when the cut-off value of Area 6–20 was set at 13.43, the sensitivity and specificity were 88.4 and 75%, respectively.

## Discussion

To our knowledge, this is the first report to explore the predictive value of heart rhythm complexity quantified by MSE analysis in chronic hemodialysis ESRD patients. The results of the present study confirm that heart rhythm complexity and cardiac autonomic function are impaired in patients with ESRD. Importantly, the deterioration of heart rhythm complexity seems to be related to a increased risk of subsequent motality. Specifically Area 6–20 among all the analyzed measures of HRV may be a predictor of increased mortality risk for all-cause death in ESRD patients. Undergoing session hemodialysis, which is complementary to existing risk stratification strategies.

The dynamic balance of ANS plays a sophisticated and important role in maintaining the homeostasis of normal physiological processes to stay healthy in the human body [26]. Since HRV measures provide information on the degree of autonomic control of the heart rate, they are often used as a non-invasive and effective tool for assessment of the cardiac autonomic function and ANS state. Furthermore, low HRV is associated with mortality in various diseases [27–30]. In the present study, we found that all of the analyzed traditional HRV parameters, as well as conventional non-linear measures and CI, were significantly different between the ESRD and healthy control groups except for the Mean RR. Consistent with previous studies [10, 31–33], lowered HRV metrics and CI were observed in our study, which confirms that ESRD patients have impaired vagal control or cardiac autonomic function as well as heart rhythm complexity caused by disease pathology. Therefore, the multiscale method showed the phenomenon of heart rhythm decomplexification in ESRD patients, which contradicting the result obtained using the traditional ApEn algorithms.

DFA is often used to quantify the fractal correlation property of the HRV time series, and the short-term fractal scaling exponent α1 has been feasible to predict mortality in various disease states [34–36]. Suzuki et al. showed that decreased α1 is an independent predictor for mortality in ESRD patients undergoing hemodialysis, and the addition of this metric to clinical risk factors significantly improves risk stratification [37]. In addition, Chiang et al. also found that increased short-term fractal scaling exponent α1is an independent predictor for lower cardiac and total mortality in patients with ESRD receiving peritoneal dialysis [36]. However, Lin et al. demonstrated that there were no significant differences in fractal scaling exponent (both α1and α2) for ESRD patients and those controls with normal renal function [38]. In the current study, we found that patients with ESRD had significantly lower α1 and higher α2 than those in healthy control subjects. This finding is consistent with previous reports of altered correlation properties under pathologic conditions [39]. Since antagonizing regulation of the sympathetic and vagal nerves on the heart is the physiological basis of the DFA method, the abnormal heart rate dynamics in ESRD patients evaluated by DFA are probably due to the co-activation of sympathetic and vagal outflow, which breakdown the intrinsic correlation property of HRV.

In this study, not only conventional non-linear measures like fractal scaling exponents and ApEn but all other traditional analyzed linear HRV metrics showed no independent predictive power for mortality in patients with ESRD receiving hemodialysis therapy. This finding is inconsistent with previous studies [9, 10, 12, 36, 37]. In addition, we observed no significant correlations of clinical risk factors with these HRV measures. The discrepancy between our findings and previous studies could be due to two main factors. The first, the ECG signals were recorded in free-running conditions, which may cause the measurements quantified by traditional linear and non-linear HRV analyses not to accurately assess the autonomic function. Additionally, different lengths of the HRV time series should also be taken into consideration for comparing the results of different studies. The second, the severity, cognitive performance, mental state and administration of drugs of ESRD patients can influence the results of HRV measures calculation, especially measurements (RMSSD, HF, α1, etc.) reflecting short-term variability and cardiac vagal regulation. The predictive ability of traditional linear and non-linear HRV parameters may be weakened and even absorbed by the associations between the above possible clinical risk factors and cardiac vagal impairment.

In contrast, heart rhythm complexity based on MSE appeared to enhance risk stratification in our study. CI Area 6–20 had the best performance for mortality prediction and is independent of clinical factors. This finding is consistent with previous studies regarding mortality using the MSE method. Norris et al. conducted a study to examine MSE parameters as predictors of trauma patient mortality in intensive care units. They concluded that MSE would be a robust predictor of trauma patient mortality in the face of variations in data density and duration [40]. The same group conducted afterward two larger studies, the results of which validated the above findings and confirmed that loss of heart rhythm complexity quantified by MSE could identify trauma patients at increased risk of subsequent hospital death [41, 42]. Ho et al. investigated the prognostic value of parameters derived from MSE in patients with systolic heart failure. They found that MSE parameter Area 6–20 showed the strongest predictive power between survival and mortality groups among all the parameters [43]. In a study in patients receiving extracorporeal life support, Lin et al. also demonstrated that MSE metrics representing heart rhythm complexity were significantly associated with mortality [44]. This evidence showed the potential relationship between risk stratification, especially mortality and decomplexification of heart rate dynamics in patients with critical illnesses. Our results indicated that not only cardiac autonomic function but heart rhythm complexity were impaired in hemodialysis ESRD patients. Furthermore, the significant association between all-cause mortality and MSE index Area 6–20 implied a direct association between ESRD outcomes and heart rhythm complexity.

There are three limitations to our study. First, our findings are based on a retrospective, single-center, small sample size of ESRD patients with different medication administration. Therefore, it is difficult to discriminate between other possible contributing factors and to predict the possible effects of drugs on the cardiac autonomic function. The predictive ability of the MSE parameters might also be underpowered due to the small sample size. Second, we analyzed ECG recordings between hemodialysis sessions. The acute and chronic effects of hemodialysis on the heart rhythm complexity of patients with ESRD might be unpredictable. Third, the endpoint of our study was all-cause mortality, whether decomplexification of heart rate dynamics is associated with cardiac death is still needs to be elucidated. Our findings are preliminary, multicenter, sizeable and prospective studies are warranted to determine the relationship between mortality and heart rhythm complexity in ESRD patients undergoing hemodialysis treatment.

## Conclusions

The present study suggests that impaired heart rhythm complexity is associated with poor prognosis in ESRD patients receiving hemodialysis treatment. Specifically, an Area 6–20 < 13.43 seems to be a significant independent and powerful predictor of increased mortality risk for all-cause death. Heart rhythm complexity also provides an additional insight into risk stratification for ESRD patients. For ESRD patients with high risk of death, regular ECG monitoring combined with antiarrhythmic drugs and subcutaneous defibrillator may be valuable and useful for managing physician in developing all-cause mortality mitigation strategies.

## Data Availability

The datasets used and/or analyzed during the current study are available from the corresponding author on reasonable request.

## Abbreviationss

ESRD: End-stage renal disease
ANS: Autonomic nervous system
HRV: Heart rate variability
MSE: Multiscale entropy
ECG: Electrocardiograms
SDNN: Standard deviation of the heartbeat intervals
RMSSD: Root-mean-square of successive differences of normal-to-normal interbeat intervals
pNN50: The percentage of normal-to-normal intervals >50 ms different from the previous interval
VLF: Very low frequency
LF: Low frequency
HF: High frequency
TP: Total power
ApEn: Approximate entropy
DFA: Detrended fluctuation analysis
EMD: Empirical mode decomposition
ROC: Receiver operating characteristic curve
AUC: Area under ROC curve
BMI: Body mass index
